# Gut Microbiota and Cardiovascular System: An Intricate Balance of Health and the Diseased State

**DOI:** 10.3390/life12121986

**Published:** 2022-11-28

**Authors:** Mujtaba Aamir Bhat, Awdhesh Kumar Mishra, Javeed Ahmad Tantray, Hanan Ali Alatawi, Mohd Saeed, Safikur Rahman, Arif Tasleem Jan

**Affiliations:** 1 School of Biosciences and Biotechnology, Baba Ghulam Shah Badshah University, Rajouri 185234, Jammu and Kashmir, India; 2 Department of Biotechnology, Yeungnam University, Gyeongsan 38541, Republic of Korea; 3 Department of Zoology, Central University of Kashmir, Ganderbal 191131, Jammu and Kashmir, India; 4 Department of Biological Sciences, University College of Haqel, University of Tabuk, Tabuk 47512, Saudi Arabia; 5 Department of Biology, College of Sciences, University of Hail, Hail 55476, Saudi Arabia; 6 Department of Botany, MS College, BR Ambedkar Bihar University, Muzaffarpur 842001, Bihar, India

**Keywords:** cardiovascular diseases, gut, gut microbiota, human health, risk factors, therapeutics

## Abstract

Gut microbiota encompasses the resident microflora of the gut. Having an intricate relationship with the host, it plays an important role in regulating physiology and in the maintenance of balance between health and disease. Though dietary habits and the environment play a critical role in shaping the gut, an imbalance (referred to as dysbiosis) serves as a driving factor in the occurrence of different diseases, including cardiovascular disease (CVD). With risk factors of hypertension, diabetes, dyslipidemia, etc., CVD accounts for a large number of deaths among men (32%) and women (35%) worldwide. As gut microbiota is reported to have a direct influence on the risk factors associated with CVDs, this opens up new avenues in exploring the possible role of gut microbiota in regulating the gross physiological aspects along the gut–heart axis. The present study elaborates on different aspects of the gut microbiota and possible interaction with the host towards maintaining a balance between health and the occurrence of CVDs. As the gut microbiota makes regulatory checks for these risk factors, it has a possible role in shaping the gut and, as such, in decreasing the chances of the occurrence of CVDs. With special emphasis on the risk factors for CVDs, this paper includes information on the prominent bacterial species (*Firmicutes*, *Bacteriodetes* and others) towards an advance in our understanding of the etiology of CVDs and an exploration of the best possible therapeutic modules for implementation in the treatment of different CVDs along the gut–heart axis.

## 1. Introduction

The human gut is colonized by microbiota of various types that hold a symbiotic relationship with the host. The entourage of gut-associated microflora forms an interactive ecosystem that plays a crucial role in the regulation of different physiological aspects and, as such, affects homeostasis between health and disease conditions [1,2,3,4]. The composition of gut microbiota varies with physiological factors such as age, dietary habits, etc., and is influenced to a great extent by external factors (probiotics, anti-microbial agents) that cause disturbance to its balance [4,5,6,7,8,9,10]. The gut microbiota forms an interactive network among the residing microbial populations, in particular bacteria that play an important role in metabolizing food constituents and absorption of essential nutrients, besides being involved in producing biologically active metabolites that, on entering systemic circulation, greatly influence the gross physiological aspects of the human body. The gut microbiota, which outnumbers host cells in the human body, is explored for its role in metabolic and immune functions and as a risk factor that increases susceptibility to diseases. Imbalance in the gut microbiome, referred to as gut dysbiosis, is associated with gastrointestinal and central nervous system (CNS) disorders, metabolic syndrome, different cancers, and the increase in risk factors associated with the development of cardiovascular diseases (CVDs) [5,6,7,8,9,10,11,12,13].

CVDs contribute to a large number of deaths worldwide, claiming more female lives than male [14,15,16]. The risk factors for CVDs include hypertension (uncontrolled blood pressure), diabetes (impaired glucose regulation), dyslipidemia and obesity, along with others such as smoking [14,15,16]. Atherosclerosis, a common cause in the development of CVDs, arises due to a series of events occurring within the arterial wall (rheology, inflammation and lipid metabolism) that progresses to arterial stenosis and, thereby, to end-organ ischemia, thromboembolic infraction and necrosis. As gut microbiota has a direct as well as indirect influence on the component risk factors associated with atherosclerosis, evidence suggests an interplay between gut microbiota and the development of CVDs [12,17,18]. Recent advances in our understanding of this connecting link made us explore the role of gut microbiota in the context of risk factors associated with CVDs, and broaden the horizons of research along the gut–heart axis toward potential clinical interventions. 

## 2. Physiological Aspects of Gut Microbiota 

**Figure 1:**
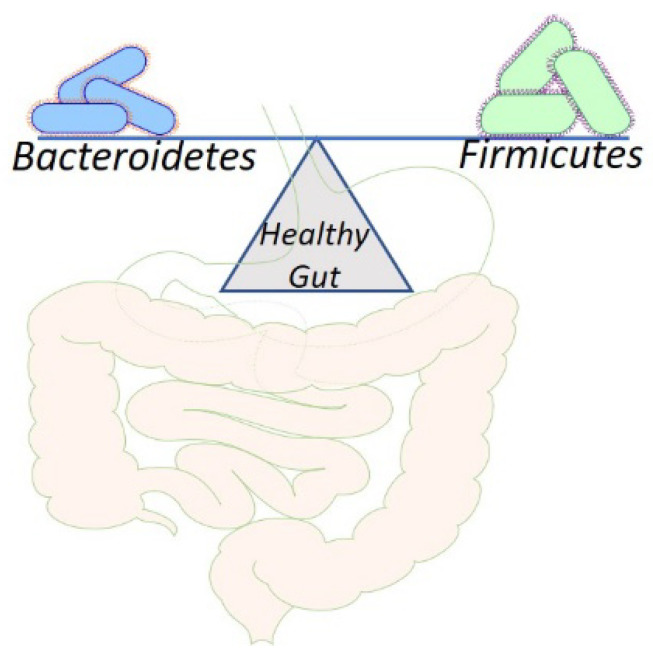
Microbiota and the healthy gut. The figure illustrates how *Firmicutes* and *Bacteroidetes* abundance helps in maintaining the balance between healthy gut and dysbiosis.

The gut microbiota (community sharing our body space) includes commensals, symbionts and pathogenic microorganisms [19,20,21]. It is believed that colonization of the gut by microbiota occurs well before delivery from maternal sources and after birth on exposure to different environmental conditions. As maternal nutrition and quality of breastfeeding dictates the microflora among newborns, milk-borne microbiota transplantation is considered critical for early onset of healthy gut microbiota [22]. Gut microbiota among newborns is distinct from other human niche populations. The mother’s milk shapes the gut microbiota of infants [23,24]. The predominant species in infant’s gut is *Bifidobacterium longum* [25,26,27]. This shows vertical transmission from mother to infant via breastfeeding. *Bifidobacterium longum*, along with other prebiotics such as galacto-oligo-saccharides, exerts broad spectrum antibiotic properties, thereby inhibiting growth of the members of Enterobacteriaceae [28,29,30]. The probiotic effect of *Bifidobacterium longum* is of transient type (declining with age), and at later stages they are replaced by anaerobic bacteria, mainly *Firmicutes*, *Bacteroidetes* and archaeal members, that persist throughout the rest of life [31,32]. The *Firmicutes/Bacteroidetes* (F/B) ratio increases from birth to adulthood, with women having high (F/B) abundance compared to men [33,34,35]. The *Firmicutes*, members of the phyla being mostly gram-positive, are capable of producing short chain fatty acids (SCFAs, particularly butyrate) that help in controlling glucose homeostasis and in improving control of blood pressure, while *Bacteroidetes* members, which are gram-negative, in addition to SCFA production (particularly acetate and propionate) are capable of polysaccharide degradation that helps in the regulation of calorie absorption [36,37,38]. As BMI significantly influences F/B abundance, it is reported that any perturbation in the proportional composition of the two provides insights into the health status of the host (Figure 1). A higher BMI (>33 group) correlates with a low F/B abundance in men compared to women, while a lower BMI value (<33 group) correlates with higher F/B abundance, in a vice versa manner [39]. Against adjustment in BMI, a higher F/B abundance gives indication of dysbiosis in the gut microbiome. In addition to abundant *Firmicutes* and *Bacteroidetes*, the gut microbial population is remarkably diverse, with a major proportion of members from *Proteobacteria*, *Actinobacteria*, *Fusobacteria* and *Verrucomicrobia* [39,40,41]. 

## 3. Dietary Pattern in Shaping the Gut Microbiota

In addition to normal physiological functions (food digestion), the gut microbiota serves as a stable yet dynamic filter for dietary exposure that produces a significant effect on the overall microbial community inhabiting the gut [42,43,44,45]. Having greater impact in shaping the gut microbiome, dietary pattern determines the diversity of gut microbial population, as macronutrients (proteins, carbohydrates and fats) induce different modulatory effects. Evaluation of the individual macronutrients for effect on gut microbiota seems problematic, as examination of one macronutrient results in alteration of the other macronutrients. Additionally, sub-categories of individual macronutrients cause dramatic differences in their effect on gut microbiota and, as such, in human health. Intake of protein of plant origin exerts severe effects on the population of *Bacteroidetes* (reduction effect) and on *Firmicutes* and lactobacillus (enhanced effect), while an opposite effect was observed on intake of proteins of animal origin, which proceeds with an increase in the tri-methylamine-N-oxide (TMAO) level [46,47]. As for a fat rich diet, deleterious effects on gut microbiota (increase in *Bacteriodetes* and *Bilophila*) are observed for saturated fats capable of inducing metabolic dysfunction, while consumption of unsaturated fats induces protection from metabolic dysfunction along with increase in the lactic acid bacteria [48]. Categorization of carbohydrates into digestible (starch and sugars) and non-digestible (fibers) is linked to different effects on the gut microbiota. Compared with the dys-biotic phenotype induced by a diet rich in digestible carbohydrates, increase in the non-digestible part increases bacterial diversity, with abundance of butyrate-producing bacteria [49]. Habitual adherence to a Mediterranean diet (rich in fiber, unsaturated fatty acids and fruits and vegetables) was found to be associated with increase in *Prevotella* and *Firmicutes*, while adherence to diet low in these components causes elevation in the circulatory TMAO levels [50,51,52]. Compared to the Mediterranean, western diet (low in fiber, but rich is saturated fatty acids and animal proteins) was found to exert negative effects on richness (microbial diversity), with decrease in beneficial species such as *Bifidobacterium* and *Eubacterium* [53,54,55]. 

## 4. TMAO as Proatherogenic Metabolite in CVD

Highlighting the essentiality of diet in determining the structure of the microbial community, diet serves as a common microbial modulator capable of affecting the gut microbial community composition and production of metabolites, preferably TMAO and SCFA (mostly acetate, propionate and butyrate). Besides acting as a signaling molecule, the gut microbiota mediated production of SFCA helps in maintaining structural integrity of the brush border epithelium [56,57,58,59]. It also helps in reducing the systolic blood pressure (an important risk factor of CVD) via decrease in the level of serum cholesterol and by improving insulin sensitivity. TMAO, casually associated with atherosclerosis, is linked to phosphatidylcholine (PC, source of TMAO in omnivores) metabolism along with choline and betaine (oxidation product of choline) [60]. The major source of dietary choline and PC is animal-based foods (red meat, dairy and eggs). TMAO is a widely recognized metabolite of choline and PC, along with L-carnitine generated by the intestinal microbiota [61]. TMAO is produced in the metabolism of choline via, intermediate trimethylamine (TMA, with the involvement of TMA lyases) by gut microbiota that undergoes hepatic oxidation, in the presence of flavin monooxygenase 3 (FMO3), to TMAO [62,63,64,65]. FMO3 (a bile activated nuclear receptor) controls the rate limiting step in the synthesis of TMAO [66,67,68]. Compared to men, women were found to exhibit higher expressional levels of hepatic FMO3 enzyme. Sex differences in hepatic FMO3 suggest hormonal regulation of FMO3 expression, as a study on castrated male mice revealed androgen dependent reduction in FMO3 expression [68]. Farnesoid X-receptor (FXR) was also found to be involved in the complex regulatory circuit that controls TMAO synthesis via FMO3 [2].

TMAO, a small sized metabolite that exhibits polar and hydrophobic characteristics, serves as an osmolyte for kidneys. High plasma concentration of TMAO results in impaired renal function (CKD, chronic kidney disease) [69], besides facilitating the development of atherosclerosis that underlies different events in the occurrence of CVD [70]. Of the different functions, TMAO is associated with modulation of the cholesterol metabolism in macrophages, hepatocytes and compartments of the enterocytes (endothelial cells lining the arterial wall) [70]. Additionally, it was also found to be involved in increasing platelet reactivity and thrombotic risk [71]. Increased amounts of TMAO in systemic circulation cause decrease in cholesterol removal, which is enhanced in its accumulation at peripheral macrophages and enterocytes, besides affecting the high-density lipoproteins in exerting their athero-protective effect [61,72,73]. TMAO in vascular cells promotes activation and, as such, enhancement in the levels of proinflammatory cytokines such as interleukin-6 (IL-6) and increased recruitment of adhesion molecules such as E-cadherin that promote inflation through the NF-kB signaling pathway [74]. Increased platelet reactivity by TMAO causes enhancement in the release of calcium (Ca^2+^) from intracellular Ca^2+^ stores that, in turn, increases thrombotic risk [71]. A strong correlation for increased risk of CVD with increase in the TMAO level was observed even after minimizing the traditional risk factors for the disease. Dietary supplementation of choline in apolipoprotein E deficient mice (ApoEˉ/ˉ) increases TMAO levels and as such atherosclerosis, while no enhancement in the plasma TMAO level was observed in germ-free (GF) ApoEˉ/ˉ [61]. Confirming the obligatory role of microbiota in the generation of TMAO, the plasma TMAO level was found to predict cardiovascular events (MI, Myocardial infraction, stroke and death), as reduction in the plasma TMAO level was found effective in preventing atherosclerosis in ApoEˉ/ˉ mice [75,76]. Together, this suggests a possible linkage between increased consumption of TMAO producing nutrients of animal origin and the increased risk of CVDs. 

## 5. Gut Microbiota in Relation to CVD Risk Factors

**Figure 2:**
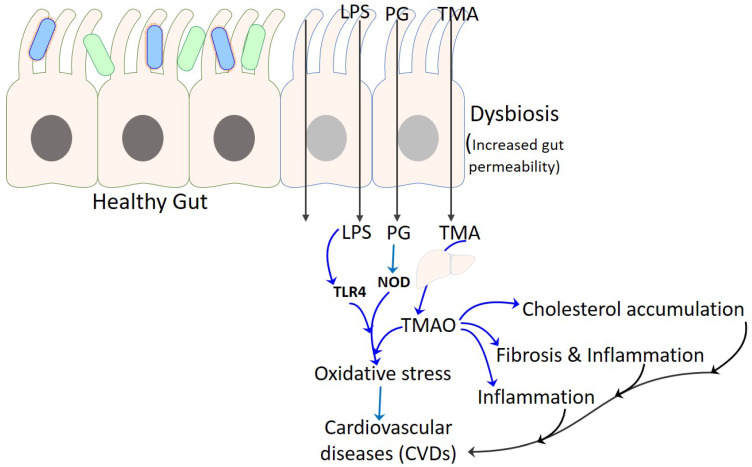
Gut microbiota and CVDs. The figure illustrates interaction of microbiota with the gut and development of different CVDs.

The current paradigm of considering metabolite TMAO as a factor in the development of CVDs provide newer insights regarding the involvement of gut microbiota (Figure 2). The following sections explore the role of gut microbial composition and associated dietary factors in exacerbating the progression of CVDs. 

### 5.1. Microbiota and Blood Pressure

On one side, where dysbiosis of gut microbiota has been implicated with the hypertensive phenotype in the host [77,78,79,80], microbial production of SCFAs were found to be exerting cardiovascular benefits [81]. In humans, the gut microbiota derived SCFAs were effective in decreasing the systolic and diastolic parameters of blood pressure [82,83]. In elucidating the relationship between microbial SCFAs and blood pressure, SCFAs exerted a blood pressure lowering effect acting through G-protein coupled receptors (GPCR41, GPCR43), while action through the olfactory receptor 78 (olf78) causes increase in blood pressure [77,81]. Acting through different receptors, the hypertensive phenotype is correlated with changes in the composition of gut microbiota, as well as in the SCFAs [84]. High-fiber diet exerts a blood pressure lowering effect through enhancement of the production of microbiota derived SCFAs [82,85]. As gut dysbiosis (altered SCFA production and increased F/B ratio) led to increased inflammation, it was found that lowering of blood pressure by SCFA is associated with reduction in the systemic inflammation via T-cell regulation and decreased aortic atherosclerotic lesions [86]. In terms of gut microbial composition, *Lactobacilli* inhabiting the gut seem most beneficial and have been linked to the anti-hypersensitive effect of blueberries, fermented milk and other dietary items [87,88]. The blood pressure lowering effect of *Lactobacilli* is partially correlated with the secretion of peptides capable of inhibiting the angiotensin converting enzyme associated with conversion of angiotensin I to a strong vasoconstrictor, angiotensin II [77,89]. This partially explains the lower blood pressure in women (prior to menopause), who have a higher proportion of *Lactobacilli* in the gut compared to men [90,91,92]. As the above findings increase the possibility of a strong association of gut microbiota derived SCFA and regulation of blood pressure, investigation on different fronts of this association is needed to understand the mechanism of operandi for its possible translation into a finite therapeutic option. 

### 5.2. Microbiota and Lipids

Gut microbiota mediated digestion of complex (non-digestible) carbohydrates and dietary fibers led to the production of different products, particularly SCFAs (abundance of acetate, propionate and butyrate) [93]. Production of butyrate is highest for guar gums, while acetate and propionate were observed in greater proportion for pine fiber and arabinogalactan [94]. Butyrate production was also observed in the fermentation of resistant starch found in food items such as rolled oats and banana flour [94]. A strong correlation was observed between SCFAs and metabolism of lipids; propionate mediated activation of receptor GPCR43 on the intestinal surface and adipose tissue was found effective in reducing lipogenesis that causes reduction in the amount of visceral and liver fat [95,96]. Having higher selectivity for GPCR43 and GPCR41, acetate and butyrate are metabolized for incorporation into fatty acids and cholesterol [56]. Interaction of SCFAs, abundant in acetate, propionate and butyrate, with peroxisome proliferator activated receptors (PPARs, a critical regulator of carbohydrate and lipid metabolism), favoring fatty acids oxidation in tissues such as liver, skeletal muscle, etc., helps in reducing lipid levels [97]. Additionally, gut microbial species, especially *Clostridium*, *Lachnospira*, *Eubacterium*, etc., capable of removing different bile acid groups (hydroxyl, taurine and glycine), yield secondary bile acids for regulating lipid metabolism (both hepatic and systemic) via FXR (bile acid receptor) [98,99,100]. Secondary bile acids interaction with FXR lowers plasma lipid concentration by decreasing serum triglycerides with simultaneous increase in the amount of HDL cholesterol [66,67,101]. Together, implication of FXR inactivation in preventing hepatic steatosis and dyslipidemia decreases the chances of occurrence of CVDs. 

### 5.3. Microbiota and Impaired Glucose Metabolism

Glycemic dysregulation (insulin resistance) that arises through dysbiosis of gut microbiota has been implicated in the development of type II diabetes [93,102]. Of the different gut dysbiosis mechanisms, one primary cause that contributes to insulin resistance appears to be low grade inflammation. As knockout Toll-like receptor 2 (TLR2) is attributed to induce insulin resistance and glucose intolerance in mice, it reveals a mechanism of disruption of insulin sensitivity by inflammation via TLR signaling cascade [103,104]. In addition to inflammation that causes modification of the gut microbiota (higher *Bacteroidetes* and *Firmicutes*, lower *Proteobacteria*), insulin resistance (in absence of TLR2) is attributed to increased lipopolysaccharides- (in serum) mediated activation of TLR4 in liver muscle and adipose tissue [105]. Serum metabolome (triglycerides, BCAAs, branched chain amino acids and membrane phospholipids) exerts a modulatory effect on insulin resistance and type II diabetes [106,107,108]. Intricately associated with different processes such as amino acid synthesis, SFCA production, bile acid transformation, etc., insulin resistance was attributed to a high proportion of *Bacteroides vulgatus* and *Prevotellacopri*, which contributes significantly to an increased number of BCAAs [19,109]. Giving credit to high serum concentration of BCAAs as a risk factor for glucose abnormality [108,110], increased activity of hepatic branched chain 2-oxaacid dehydrogenase (BCODH) that enhance catabolism of BCAA plays an important role in attributing a protective effect for insulin resistance and type II diabetes. 

### 5.4. Microbiota and Obesity

A bidirectional relationship between obesity and gut microbiota is reported. In this intricate relationship where obesity is capable of altering the gut microbiota composition, gut microbiota in return regulates important events (energy extraction and expenditure) in the etiology of obesity [39,111,112,113,114,115]. As dietary intake modulates the composition of gut microbiota, consumption of fiber-rich diet favors lower F/B ratio with increased proportion of *Bacteroidetes* [116,117]. A higher proportion of *Firmicutes* in F/B ratio observed among overweight and obese persons indicates more calorie extraction from food that proceeds to the development of obesity [112,113,114,115]. Alongside this, breakdown of otherwise indigestible polysaccharides increased the amount of LPS endotoxin release into circulation, thereby having a greater influence on fat storage and adipose tissue inflammation that proceeds to the development of obesity [112,118]. The SCFAs produced by gut microbiota activate the lipogenic enzymes such as sterol response element binding protein 1 (SREBP-1) in liver that promotes enhanced storage of triglycerides [119]. Additionally, suppression of the expression of fasting induced adipocyte factor (FIAP, an inhibitor of lipoprotein lipase) promotes fat storage and, as such, development of an obese phenotype [119,120]. The interplay between gut microbiota mediated SCFAs production and obesity needs further elucidation in establishing the role of gut microbiota in preventing the development of the obese phenotype. 

## 6. Gut Microbiota and Cardiovascular Diseases

CVDs including hypertension, atherosclerosis, etc., produce an immense health and economic burden in the global context. It is considered as the leading cause of deaths in the developed nations. In addition to alteration in the composition of gut microbiota, the metabolic potential of gut microbiota is identified as a potential contributor to the development of CVDs. The following sections deals with the study of interaction of the gut microbiota with the host that proceeds to the development of different CVDs. 

### 6.1. Hypertension

Hypertension, a multifactorial disorder related to genetic and environmental factors, affects over 1 billion individuals worldwide [54]. Considered as the most important risk factor associated with the development of CVDs, its occurrence is commonly correlated with dietary factors, obesity and stress. There is mounting evidence that suggests a possible link between alteration of the gut microbiota and elevation in the levels of blood pressure (BP) [121,122,123]. The first evidence of this phenomenon, linking gut microbiota to regulation of the BP, was provided in the studies of attenuation of steroid induced hypertension following antibiotic treatment [124,125,126]. In another study, Shikata et al. [127] observed significant reduction in the incidence of hypertension related aneurysm on ablation of the gut microbiota following antibiotic dose. When comparing microbiota of normotensive and hypertensive rats, Yang et al. [128] reported significant reduction in the occurrence of microbiota (richness, diversity, etc.) with an increase in the F:B ratio. The concept of reduction in microbiota richness with increase in the F:B ratio among affected hosts is corroborated by data from several studies performed on hypertensive models [82,129]. These changes are attributed to reduction in the acetate and butyrate production among *Bacteroidetes* and *Firmicutes*, respectively, that contributes significantly to high BP [130]. Pointing to a strong correlation of microbiota dysbiosis with the hypertensive phenotype, the F:B ratio is considered as a strong parameter in determining the metabolic composition of the microbiota of an individual. Fecal transmission from hypertensive to normotensive mice revealed an elevated BP, while vice versa was insufficient in normalizing microbial dysbiosis and thereby reducing the levels of BP [128,131]. 

The linking mechanism in this connection includes effects mediated by a large number of vasoactive metabolites (TMAO, norepinephrine, etc.), along with an involvement of SCFAs and systemic inflammation [132]. A study performed on TMAO with respect to its role in hypertension revealed that enhancement in TMAO levels has a propensity to elevate BP [131]. The levels of bacterial metabolites, referred to as SCFAs, on fermentation of dietary fiber in the colon have direct involvement in regulating the hypertensive phenotype via interaction with the G-protein coupled receptors (GPCRs, primarily Gpr41 and Olfr78) [133]. In elucidating the role of SCFAs in hypertension, it was observed that activation of Gpr41 contributes to the hypotensive phenotype, while increase in the stimulation of Olfr78 led to elevated levels of BP. In a study elucidating the effect of intake of a high salt diet (HSD), Wilck et al. (2017) observed that HSD mediated elevation of BP is accompanied with reduction in the levels of *L. murinus* and replenishment of its levels prevented development of the hypertensive phenotype via inhibition in the induction of T-helper (TH)-17 cells [123]. Additionally, HSD is often related to changes in the level of microbial metabolites such as 4-ethylphenylsulfate (a metabolite linked to benzoate metabolism) in the gut [134]. In another study, rats fed on HSD HF higher levels of acetate and propionate (characteristic of *Bacteroidetes*) compared to their control [135]. In addition to the above mentioned factors, Li et al. reported that a high percentage of the occurrence of bacteria of genus *Prevotella* also contributes to the hypertensive phenotype [121]. Furthermore, application of antihypertensive drugs in reducing the BP was found to act through regulation of the gut microbiota. A drug, Captopril (Cap, an inhibitor of angiotensin converting enzymes) was found to increase the levels of *Allobaculum* and was capable of maintaining a sustained antihypertensive effect, even on withdrawal of the CAP [136]. A similar antihypertensive effect was observed for the drugs Candesartan and Irbesartan, which prevent the disruption of gut microbiota by preserving *Lactobacillus* levels and as such normalizes the F:B ratio [137,138,139]. 

### 6.2. Atherosclerosis

Atherosclerosis is a chronic inflammatory disease characterized by attack on the endothelial lining of the arterial wall followed by lipid accumulation and finally by recruitment of the macrophages and other immune cell types [140,141]. It generally remains asymptomatic until narrowing of arteries reaches thrombotic occlusion, thereby causing insufficient flow of blood and, as such, deprivation of oxygen in the heart muscles [142,143]. Marked by the presence of plaques (atheromas), it exerts its effects on both large and small sized arteries, which progresses to different kinds of vasculopathies. Atherosclerosis involves components of both metabolic and inflammatory systems that are influenced to a large extent by alterations of the gut microbiota. Evidence of microbial contribution to development of atherosclerosis was first reported in the studies on atherosclerotic plaques having DNA of different bacterial species [144,145,146]. In a study on atherosclerotic plaques, Koren et al. reported that presence of *Chryseomonas* in all and the presence of *Streptococcus* and *Veillonella* in the majority of cases of atherosclerosis [147]. The authors observed that bacterial phylotypes observed on the atherosclerotic plaques display similarity with those present in the oral cavity and gut of the same individual. The results highlighted a possible contribution of gut microbiota (remote bacterial communities) to the development of atherosclerosis. A study of the microbial profile of patients with CVD following employment of the terminal restriction fragment length polymorphism method revealed an enhancement in the *Lactobacillales* and *Clostridium* level with a subsequent reduction in the levels of *Bacteroides* [148]. High abundance of *P. gingivalis* and *Actinomycetem comitans* in the gut also contributes to their abundance in atheromas [147].

Gut dysbiosis altering the production of microbiota derived metabolites also exerts pro-atherosclerotic effects in the host. As TMAO is considered as the major contributor in the development of atherosclerosis, inhibition in the production of microbial TMA attenuates induction in the development of pulmonary artery atherosclerosis in an apnea-associated obstructive sleep mouse model [149]. Microbiota generated metabolites such as butyrate exhibiting an athero-protective effect indicates an important role of the gut microbiota metabolism with respect to development of atherosclerosis [150,151]. Dysbiosis associated disturbance in the gut microbiota composition that increases intestinal permeability led to elevation in the levels of circulating LPS. Sensing of the LPS by TLR-4 receptor promotes inflammation and foam cell formation via induction in the downward signaling cascade mediated by MYD88 [152]. TMA being an important precursor in the formation of TMAO via carnitine and choline specific lyases, inhibition of the enzymatic system was effective in reducing the atherosclerotic lesions in ApoEˉ/ˉ mouse model [75]. Being an important player in the development of atherosclerosis, enhancement in the circulating TMAO level was effective in modulating the platelet hyperreactivity (a phenomenon of activation, adhesion and aggregation of platelets) that progresses with the formation of thrombi [71,153]. TMAO is used as a prognostic marker in detecting the vulnerability to the development of coronary plaque that increases the risk for CVD-related problems [76,121].

### 6.3. Cardiomyopathy and Heart Failure

Representing an end stage of different CVDs, heart failure includes a series of complex clinical complications that affects the structure and functionality of the heart [154,155]. In the recent past, a concept emerged regarding the role of gut microbiota’s attribution to the pathogenesis of heart failure, referred to as the “gut hypothesis of heart failure”. According to this hypothesis, an increase in systemic congestion followed by a decrease in cardiac functioning (cardiac output) led to an increase in the translocation of bacteria and, as such, to circulating toxins, which is the underlying primary cause of the inflammation among patients with heart failure [156,157]. The intestinal hyper-fusion caused by reduction in the cardiac output induces ischemia or edema of the intestinal mucosa, which progresses with impairment to the barrier functioning, and thereby exacerbates the development and progression of heart failure via enhancement of the translocation of gut microbiota along the cardiovascular axis [158,159]. Biopsic study of colonic mucosa have revealed that patients with lower levels of intestinal blood flow exhibit higher serum levels of anti-LPs IgA, which is correlated with enhancement of the growth of bacteria [160]. Chronic heart failure patients were found to be colonized primarily by different pathogenic bacteria (*Yersinia enterocolitica*, *Campylobacter*, *Shigella*, etc.) along with some fungal isolates such as *Candida* [161,162]. These bacterial and fungal isolates are positively correlated for their contribution to the severity of heart failure. The bacteria were found more abundantly in decompensated than to compensated heart failure cases over a short period of time [163]. In another study, a short diuretic treatment was found to be effective in decreasing serum endotoxin levels [164].

Development of heart failure is influenced to a greater extent by differences in the gut microbiota composition via alteration of the gut microbiota metabolite profile. Compared to healthy controls, serum TMAO levels were found to be higher in patients with heart failure of the same age and gender [165]. Higher levels of TMAO among heart failure patients were associated with higher mortality (~1.18 fold) and cardiac transplantation rates (~1.79 fold) [166]. Enhanced serum TMAO level has a remarkable value as a prognostic marker in heart failure risk stratification [61,165,167,168]. The microbiota generated TMAO and supplementation of choline and TMAO via diet increase TMAO levels in the systemic circulation, which contributes significantly to enhancement of myocardial fibrosis, besides having a severe effect on parameters controlling transaortic constriction induced heart failure [159]. It is believed that TMAO increases susceptibility to the development of heart failure via an increase in ventricular remodeling (by prolonging the angiotensin effect) and myocardial fibrosis (activating NLRP3 inflammasome signaling along the TGF-/Smad3 cascade), accompanied by cardiac dysfunction [169,170]. Additionally, gut microbiota metabolites other than TMAO were also found to exert their effect on the metabolism of bile acids, as their composition was altered in patients with heart failure [171]. Their effect on BA metabolism was found to alter primary (decreased) and secondary (increased) bile acid levels among patients with chronic heart failure. Propionate significantly reduced the occurrence of fibrosis and cardiac hypertrophy, besides being capable of attenuating vascular dysfunction via regulation of the T-cell function [86]. 

## 7. Gut Microbiota Interventions for Cardiovascular Diseases

Having sufficient evidence that shows a strong link between gut microbiota composition, metabolites and susceptibility to cardiovascular pathophysiology has put the spotlight light on gut microbiota as a novel regulator in the occurrence and development of CVDs. It has also highlighted the need for microbiota targeted therapies capable of altering the gut microbiota composition and the production of different metabolites as therapeutic interventions in avoiding the development of CVDs. A large number of approaches, such as dietary interventions, use of pre-, pro- and post-biotics along with the design of small molecule inhibitors, are currently being employed in the manipulation of gut microbiota with promise in avoiding the occurrence of CVDs. The following sections deal with elaborations on therapeutics tools for CVDs.

### 7.1. Dietary Interventions

Dietary intervention-based modulation of the gut microbiota was observed as an effective strategy in reducing the risk of CVDs [172,173]. Though the gut microbiota composition remains resilient during an individual’s life span, changes in nutrients brought about by dietary interventions result in modifying the composition of resident gut microbiota [46,174]. Changes in the carbohydrate content in the diet were found to be effective in altering the composition of *Roseburia* and *E. rectale* [175,176]. Higher consumption of a fiber rich diet was associated with an increase in acetate producing bacteria, besides being effective in lowering the BP in hypertensive patients and decreasing the risk of coronary diseases and the chances of cardiac hypertrophy and fibrosis [82,177,178]. Where a fiber rich diet promotes the growth of beneficial commensal microbiota, it was found to be effective in reducing the growth of opportunistic pathogens [179]. Consumption of traditional Chinese medicine along with whole grains and sufficient prebiotics were effective in reducing the levels of the members of *Enterobacteriaceae* (often designated as opportunistic pathogens), with concomitant increase in the members of the *Bifidobacteriaceae* family, with a definite role in extending protection to gut mucosa [180]. The Mediterranean diet was effective in reducing the occurrence of CVDs via reduction in the levels of TMAO [173,181]. Inhibition of gut microbiota-dependent TMAO production has been a promising strategy for the treatment of atherosclerosis [182]. As part of improving an individual’s health, supplementation with ginger is effective in altering the expression of genes that undergo interaction with the host, besides modulating the composition of gut microbiota and inducing an increase in fatty acid metabolism [183,184]. Zinc (Zn, an essential trace element serving as co-factor to bacterial proteins) also alters the gut microbiota composition [185]. Capable of altering the microbiota more towards β-diversity, this shift in turn aggravates Zn deficiency conditions [186]. Dietary supplementation of zinc along with curcumin was effective in improving gut dysbiosis caused by Zn deficiency and exerts a protective effect against doxorubicin induced cardiotoxicity in rats [138]. A dose-dependent effect was observed for Zn supplementation on gut microbiota composition, with an excess amount associated with an increase in intestinal permeability which enriches the gut with pathogenic microbial species [187,188].

### 7.2. Prebiotics and Probiotic Interventions

Prebiotics represent a large and diverse set of substances of non-microbial origin such as fibers, minerals (Zn, Fe, etc.), vitamins (A, B1, B6 and others) and a complex series of saccharides, capable of eliciting a favourable impact on the abundance (and regulating the composition and functioning) of gut microbiota that confer benefits to the host [189,190]. Capable of escaping the effects of pancreatic enzymes, they hold the potential to serve as a source of energy for colonic bacteria [191]. Resisting metabolization by the gut microbiota, it holds the ability to selectively alter its composition. Intake of oligofructose causes an increase in the number of beneficial bacteria such as *Bifidobacterium* and *Lactobacillus*, with significant reduction in intestinal permeability and inflammation of the hepatic system [192,193]. In obese patients, oligofructose supplementation improved glucose tolerance, besides controlling and regulating the plasma lipid profile [194,195,196]. Inulin supplementation induced a significant reduction in the occurrence of atherosclerotic lesions in an ApoEˉ/ˉ mouse model [197]. It was found that prebiotics reverse the features of gut microbiota, with significant improvement in gut permeability and inflammation, which improved glucose intolerance. 

**Figure 3:**
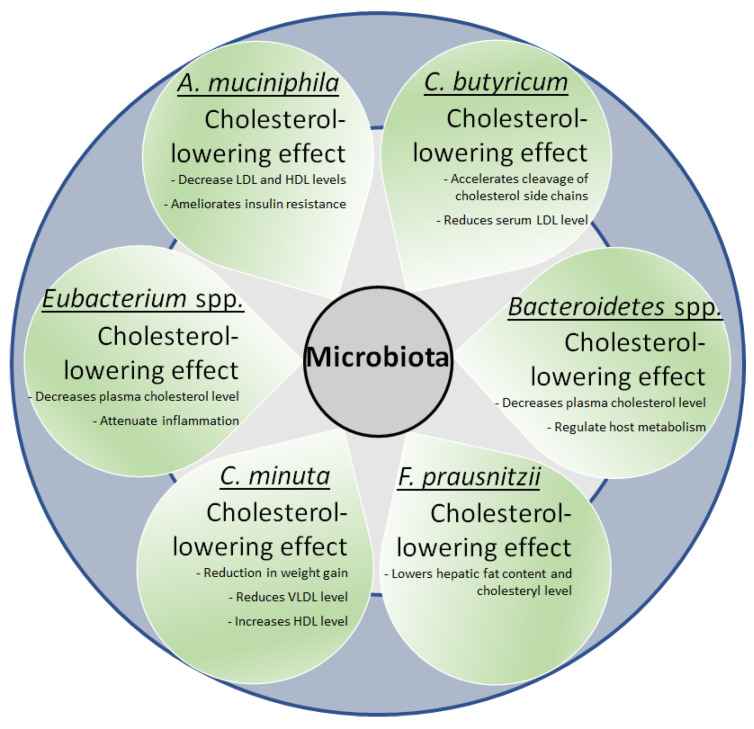
Microbiota supplementation in gut and CVDs. The figure illustrates positive effects observed on supplementing different bacteria in the gut regarding risk factors associated with CVDs.

Probiotics are categorized as bacteria that, on administration in the host, help in re-establishing an ecological niche in the intestines for maintaining the balance of beneficial bacterial species via modulation in the pH, regulation of the production of antibacterial compounds, and in exerting a competition against the establishment of pathogens [198]. A large number of bacterial species such as *Bifidobacterium*, *Faecalibacterium*, etc., have been used to correct the gut microbiota towards restoration of innate and adaptive immunity [199,200]. Administration of *Lactobacillus rhamnosus* GR1 improved cardiac functioning and induction of *Saccharomyces boulardii* caused improvement in the left ventricular ejection fraction [201,202]. Administration of Lactobacillus sp. effectively reduced the level of toxins such as dimethylamine produced by the small intestines of patients suffering from CVDs, besides reducing the levels of certain SCFAs in individuals suffering from carotid atherosclerosis [203,204]. Administration of *L. reuteri* promotes release of incretin that increases insulin secretion, as observed in obese glucose-tolerant subjects [205]. Atherosclerosis was attenuated on treating ApoEˉ/ˉ mice with *L. rhamnosus* GG, *L. acidophilus* ATCC 4358, *A. muciniphila* and other probiotics [206,207,208] (Figure 3). Li et al. [206] reported reduced levels of *A. muciniphila* in mice susceptible to atherosclerosis and observed a reversal in its effect on exogenous replenishment of *A. muciniphila* [206]. A large number of probiotics were effective in lowering BP in the hypertension models [209,210]. In another study, daily consumption of ≥10^9^ CFU of probiotics was effective in reducing systolic as well as diastolic levels in patients suffering from hypertension [83]. Apart from these beneficial effects, it is believed the administration of probiotics to patients with weak immunity might cause endocarditis on becoming converted to opportunistic pathogens [211], indicating the need for a careful examination of vulnerable groups of patients before administration of probiotics.

### 7.3. Fecal Microbiota Transplantation

FMT is an alternative therapeutic intervention approach used in the treatment of patients with dysbiosis. In this process, a fecal microbial population from healthy individuals is transplanted to the gastrointestinal tract for displacement of pathogens and subsequent resumption of normal functioning of the gut microbiota [212,213]. The procedure has mainly been employed in the treatment of intestinal diseases including that caused by *Clostridium difficile*, where it was found effective in reducing (~80%) the infection rate [214,215,216,217,218,219]. Despite this, the use of FMT in the treatment of dysbiosis via a bile acid dependent mechanism received only limited approval from the FDA due to a recurrence rate of ~25–30% [220,221,222]. Of the different studies that examined the effect of FMT on cardio function, transfer of FMT was found effective in rebalancing the microbial community composition and, as such, in the attenuation of myocarditis-associated myocardial injury in an autoimmune myocarditis mouse model [223]. The FMT approach was effective in reducing the chances of development of atheroma and preventing inflammation via reduction in the infiltration of macrophages [206]. Though an FMT approach was effective in improving insulin sensitivity in males with metabolic syndrome, the effect was transient as no significant improvement was observed at 18 weeks after transplantation [102,224]. Fecal transplantation increases the abundance of butyrate-producing bacteria, preferably *Roseburia*, which plays important role in maintaining glucose level. Additionally, studies have also pointed towards possible transmission of obesity, IBD and other conditions including susceptibility to atherosclerosis via TMAO and endotoxemia in ApoE-knockout mice [225,226,227]. With the threat of transfer of endotoxins and other infective agents during FMT that increases the chances of infection [228,229], it becomes obligatory to further investigate the possible risks of adverse effects and devise a proper strategic management program for improving its efficiency, and as such its applicability for use in the treatment of cardiovascular disorders. 

### 7.4. Small Molecule Inhibitors

With the discovery of the involvement of TMAO as a hallmark of CVD outcome, studies were directed to selectively targeting the enzymes responsible for the production of TMAO or its precursor TMA as part of an effective potential therapeutic strategy. The concept behind this strategy was to develop potential inhibitors with properties to inhibit bacterial choline TMA lyase [75]. DMB (1,3 dimethyl-1-butanol), a structural analog of choline, was effective in reducing plasma TMA and TMAO levels in mice fed on choline or carnitine diet, thereby contributing significantly to prevention of the development of aortic root atherosclerosis [75,149]. DMB mediated inhibition of TMA production was found effective in reducing TMAO levels without having any impact on the growth of microbial commensals, thus improving hemodynamic parameters [230]. Additionally, exogenous introduction of alkaline phosphatase was effective in detoxifying bacterial toxins such as LPS, thereby producing a pronounced effect in preventing metabolic syndrome and HFD induced endotoxemia in mice [231]. Being effective in reducing the chances of gut dysbiosis, it uncovers a precise mechanism towards development of effective therapeutic interventions against different CVDs.

## 8. Conclusions and Future Perspectives

The human gut microbiota is comprised of non-pathogenic commensal microorganisms that exhibit extensive metabolic capabilities. With the ability to influence efficiency in harvesting energy from the diet, they aid in the digestion and absorption of nutrients in the gut. Accumulating evidence suggests a significant contribution of gut microbiota in the occurrence and pathogenesis of different diseases, including the CVDs that serve as a leading cause of death among men and women worldwide. A series of risk factors such as hypertension, diabetes, dyslipidemia, etc., were found to play an important role in the development of CVDs. The risk factors (high blood pressure, impaired glucose metabolism, obesity) associated with CVDs were influenced by the composition of gut microbiota, suggesting a possible interplay between microbiota and the development of these CVDs. A transient shift from *Bifidobacterium longum* in infancy to *Firmicutes*, *Bacteroides* and other archaeal members in adulthood was often associated with regulation of gut microbiota composition and the production of metabolites such as TMAO and SCFA. While TMAO is associated with the modulation of cholesterol metabolism, SCFA helps in maintaining the structural integrity of the brush border epithelium, besides playing an important role in reducing systolic blood pressure via improvement in insulin sensitivity (glucose metabolism) and a decrease in serum cholesterol level. 

There are several constraints associated with the correlation of microbiota and CVDs. The first involves change in the microbial population and its dynamics with respect to time. All we have is information at the first instance and during the course of time in which the microbiota composition changes and, as such, changes function. Secondly, the microbiota composition exhibits variation across different population groups and via the dietary pattern. The exact composition of the microbiota associated with a healthy individual changes with respect to dietary pattern. Thirdly, a large number of factors such as BMI, age, gender, etc., influence the abundance of microbiota. Lastly, information about the exact types of microbial population that define the gut microbiota is still missing, as only few groups have been explored and studied with respect to their role in healthy humans and persons suffering from different diseases. Despite these limiting factors, gut microbiota is believed to have serious implications in the management of different diseases, including CVDs. 

The gut microbiota needs further characterization in terms of its role and a deep exploration is required for the production of different metabolites that are thought to have a role in mitigating the development of different CVDs. As modulation of the dietary intake, supplementation with pre- and probiotics is effective in reducing the risk of CVDs, opening up new avenues in directing studies towards development of novel therapeutics, including small molecule inhibitors, that can be employed in modulating the composition of gut microbiota and leading to a reduction in the development of CVDs. Studies are also needed to gain insight in to risk control measures via identification of the microorganisms exhibiting potential benefits in achieving a precision series of probiotics for employment in the treatment of different CVDs. As microbiota-based therapies are sparse, further studies are needed that could advance our knowledge and understanding of the underlying mechanisms, with a view to translating the benefits of microbiota in clinical settings towards the development of realistic solutions in controlling the occurrence of different CVDs. 

## Data Availability

Not applicable.

## Abbreviations

CVD, Cardiovascular diseases; CNS, Central Nervous System; F/B, *Firmicutes*/*Bacteroidetes*; SCFAs, Short Chain Fatty Acids, BMI, Body Mass Index; TMAO, Trimethylamine-N-oxide; PC, Phosphatidylcholine; FMO3, Flavin monooxygenase-3; FXR, Fernesoid X-receptor, CKD, Chronic Kidney Disease; APOE, Apolipoprotein E; GF, Germ free; MI, Myocardial infraction, GPCR, G-protein coupled receptor; TLR, Toll-like receptor; BCAA, Branched chain amino acid; BCODH, Branched chain 2-oxacid dehydrogenase; HDL, High density lipoprotein; LPS, Lipoprotein; FIAP, Fasting induced adipocyte factor; HSD, High salt diet; FMT, Fecal microbiota transplantation; DMB, 1,3-dimethyl-1-butanol; HFD, High fat diet.
